# Practical health co-operation - the impact of a referral template on quality of care and health care co-operation: study protocol for a cluster randomized controlled trial

**DOI:** 10.1186/1745-6215-14-7

**Published:** 2013-01-07

**Authors:** Henrik Wåhlberg, Per Christian Valle, Siri Malm, Ann Ragnhild Broderstad

**Affiliations:** 1 Department of Community Medicine, Faculty of Health Sciences, University of Tromsø, 9037, Tromsø, Norway; 2 University Hospital of North Norway Harstad, St. Olavsgate 70, 9480, Harstad, Norway; 3 Centre for Sami Health Research, University of Tromsø, 9037, Tromsø, Norway

**Keywords:** Cluster randomized trial, General practice, Quality of care, Referral

## Abstract

**Background:**

The referral letter plays a key role both in the communication between primary and secondary care, and in the quality of the health care process. Many studies have attempted to evaluate and improve the quality of these referral letters, but few have assessed the impact of their quality on the health care delivered to each patient.

**Methods:**

A cluster randomized trial, with the general practitioner office as the unit of randomization, has been designed to evaluate the effect of a referral intervention on the quality of health care delivered. Referral templates have been developed covering four diagnostic groups: dyspepsia, suspected colonic malignancy, chest pain, and chronic obstructive pulmonary disease. Of the 14 general practitioner offices primarily served by University Hospital of North Norway Harstad, seven were randomized to the intervention group. The primary outcome is a collated quality indicator score developed for each diagnostic group. Secondary outcomes include: quality of the referral, health process outcome such as waiting times, and adequacy of prioritization. In addition, information on patient satisfaction will be collected using self-report questionnaires. Outcome data will be collected on the individual level and analyzed by random effects linear regression.

**Discussion:**

Poor communication between primary and secondary care can lead to inappropriate investigations and erroneous prioritization. This study’s primary hypothesis is that the use of a referral template in this communication will lead to a measurable increase in the quality of health care delivered.

**Trial registration:**

This trial has been registered at ClinicalTrials.gov. The trial registration number is NCT01470963

## Background

Quality of healthcare is defined by the American Institute of Medicine as ‘the degree to which health services for individuals and populations increase the likelihood of desired health outcomes and are consistent with current professional knowledge’ [1]. The focus on prevention of medical errors and improved quality of medical care continues to increase [2,3]. This focus is evident in Norway by the publication of a national strategy for quality improvement in health and social services [4], and the multitude of clinical guidelines available from governmental and professional sources.

The referral of a patient from a general practitioner (GP) to a hospital environment represents a transition of care, in which the major information exchange is through the written referral letter [5]. This transition of care represents an important step in the quality of the care process, and it has been shown that key clinical information may not be communicated adequately at the transition of care interface [6]. There has been considerable research into the quality of a referral and its impact on the process of care. A Norwegian study from 2007 amongst elderly patients demonstrated that both referral and discharge letters were missing vital information [7]. The consequences might represent a health hazard to older patients [7]. A Finnish assessment of the quality of referral letters for patients with asthma concludes that 45% of the referrals were of poor or unacceptable quality, based upon quality criteria developed by GPs and hospital respiratory consultants [8]. Others have also found varying quality and content of referrals [9-15].

Many studies have been designed for improving the referral process from GPs to the hospital [16,17]. A recent Cochrane review on interventions to improve outpatient referrals from primary to secondary care concludes that surprisingly few interventions on the referral system have been rigorously evaluated. Many of the studies evaluated focused only on referral rates or referral quality. The review highlights the complexities of research in this area, especially as no single study managed to present findings on all aspects of the referral process (referral behavior, management of non-referred patients, secondary care management of patients, the flow of patients through the referral system, patient outcomes and satisfaction, and resource use). However, structured referral sheets and local education interventions have an impact on referral rates [18].

The primary objective of the present study is to evaluate whether the implementation of a referral template in the referral from general practice to hospital care can lead to a measurable increase in the quality of care delivered at the hospital. As secondary objectives, we will assess patient satisfaction and effective prioritization at secondary care.

## Methods

### Study design

This study is a cluster randomized trial, with the GP clinics as the cluster. The local GP clinics are randomly assigned either to use a referral template or to continue standard referral practice.

### Participants

The 14 community GP practices in the area primarily served by University Hospital of North Norway Harstad (UNN Harstad) were included in the randomization process, with a total list size of 35,490 patients. In Norway, each individual has a regular GP. These GPs act as gatekeepers to secondary care. The health care system is relatively uniform throughout the country. In the study area, access to specialist care is practically impossible without a GP referral, whereas some access is possible in other areas of the country.

The study population will consist of patients referred to the medical department at UNN Harstad. The referrals received are, almost exclusively, electronic. Children (<18 years of age) and patients with reduced capacity to consent will be excluded from participation in the study.

### Randomization

**Figure 1:**
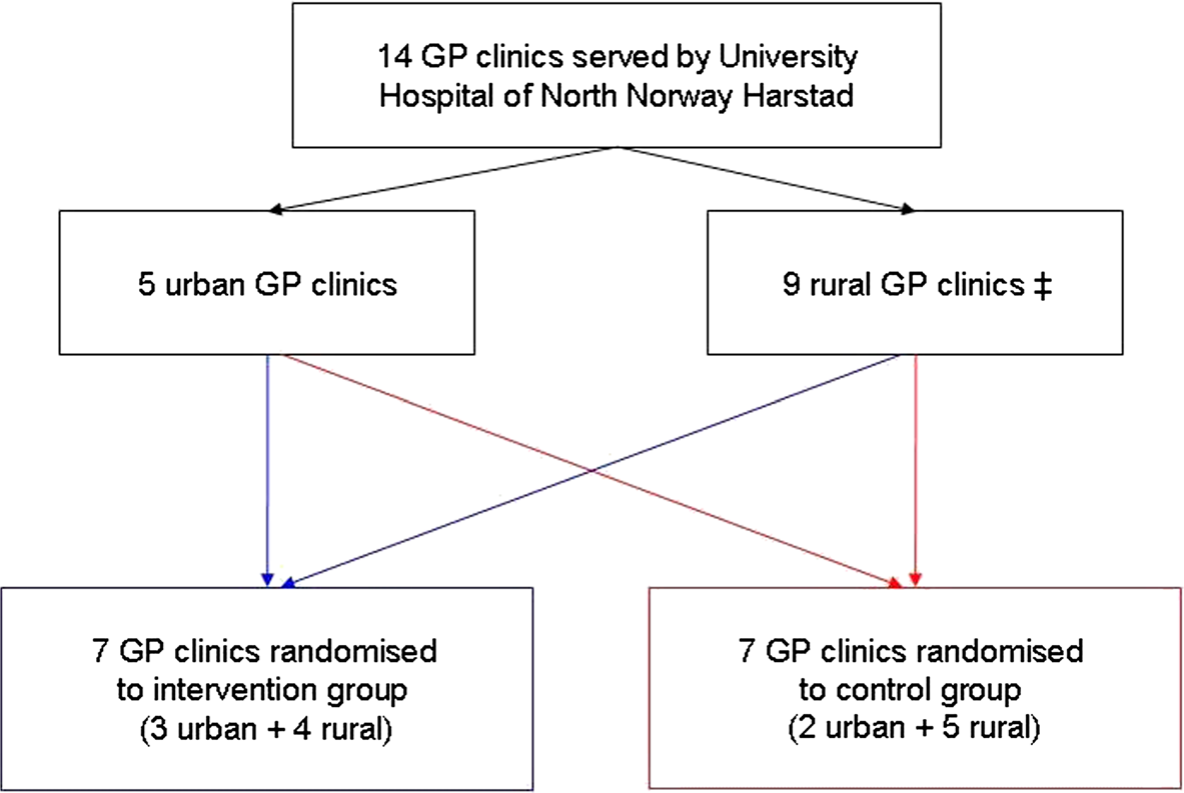
**Flow chart of randomization process.** ‡ From the four rural clinics initially randomized to the intervention group, two clinics refused. Therefore two additional rural clinics, from the five rural clinics initially randomized to the control group were randomized, and consented to, the intervention.

The GP clinics were randomized stratified by location, to ensure adequate selection of cases and equal sociodemographic background data. Five of the centers are larger town-based centers and nine are smaller, more rural centers. The location of the center was not expected to influence the outcome variables. Initially, two centers approached declined the invitation to participate in the study, and therefore two additional GP clinics were randomly selected, as illustrated by Figure 1.

### Recruitment

**Figure 2:**
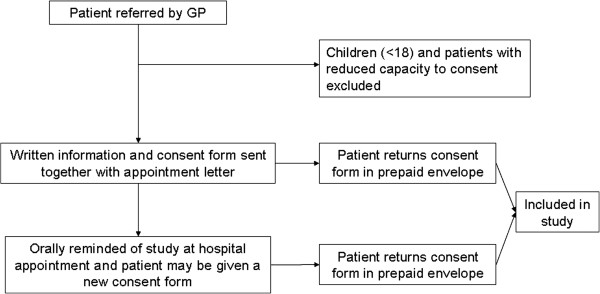
**Recruitment process.** Recruitment of patients in both the intervention and control groups follow the same procedure.

New patients referred to the medical department within one of the four diagnostic groups described below will receive written information and a consent form together with their appointment letter. These will be sent out by a clinic nurse unaware of the status of the GP center sending the referral (intervention or control). Patients will be orally reminded at the appointment and may be given a new consent form. This process is illustrated in Figure 2.

### Intervention

The referral templates have been developed based upon international literature [19-27] and in collaboration with local specialists in the appropriate medical field. The templates have also been through a process of clinical assessment from subspecialists in other northern Norwegian hospitals. In acknowledging the problems in earlier studies with the uptake of referral interventions [17,18] we have deliberately reduced the number of items in the referral templates, to ensure ease of uptake. Only information that the medical consultants thought imperative in the referral have been included as items in the templates. The study will implement referral guidelines for the following four diagnostic groups:

• patients referred with dyspepsia;

• patients referred with suspected colonic malignancy;

patients referred with chest pain;

• patients referred with chronic obstructive pulmonary disease or suspected chronic obstructive pulmonary disease.

These diagnostic groups were chosen as they represent a substantial number of the referrals to a medical department (own data, 2008). They also represent a clear diagnostic challenge in primary care and are adept for simple referral guidelines.

**Figure 3:**
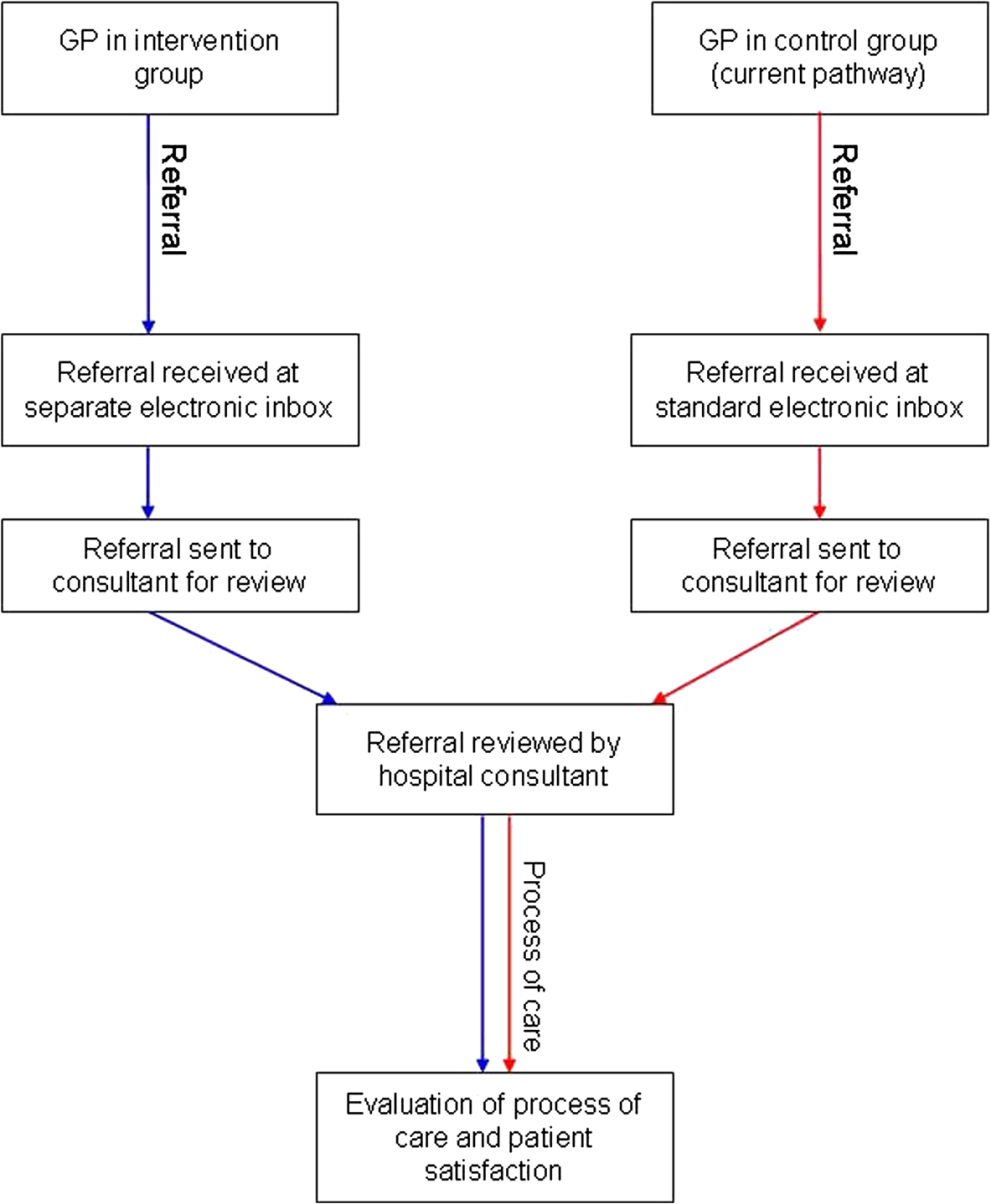
**Referral pathway.** Flow of referral and process of care in the intervention (blue arrows) and control group (red arrows).

The GPs at the intervention offices will use the referral template when initiating a new referral process for a patient. To ensure adequate uptake of intervention, the templates have been distributed as electronic templates as well as hard copies. The templates function as guidelines, but are not implemented as compulsory electronic checklists. The intervention referrals are sent to a separate electronic inbox at the hospital. The further evaluation and process of care has not been altered in the intervention group compared with the standard referral practice in the control group (Figure 3).

In addition, questionnaires have been developed that assess patient experiences within the care framework, with one questionnaire designed for inpatients and one for outpatients. These questionnaires have been produced by combining questions from previously validated questionnaires regarding patient experiences in general and with transitional care. They include questions from a Norwegian patient experiences questionnaire [28]); two questions about health interaction from the Commonwealth Fund Survey 2010 [29]; the Care Transitions Measure 3 [30]; and demographic questions.

The questionnaires have been reviewed through an interview process with patients. This was done to ensure that the forms are acceptable to patients and to highlight possible issues that patients felt were missing from the questionnaires.

### Objectives

We aim to examine the impact of a referral template on the process of care, at the individual level. The primary hypothesis is that the use of a referral template in the communication between the GP and secondary care will lead to a measurable increase in the quality of health care delivered. Secondary hypotheses include that the use of a referral template in the communication between a GP and secondary care will lead to:

a • measurable improvement in referral quality;

• a change (up or down) in the number of patients defined as being in need of prioritization (as defined in national guidelines for prioritization in health care [20,23,24]);

• more appropriate prioritization, as measured by final diagnosis;

• an increase in the ‘appropriateness’ of the referrals (positive predictive value (PPV) of referral [31];

• and better patient satisfaction, as measured by self-report questionnaires.

### Outcomes

The primary outcome will be a quality indicator score compared between the intervention and the control group. The quality indicators have been generated from previous international quality assessment tools and national and international treatment guidelines. Some adaptation to locally accepted practice has been made, as demonstrated by others when quality indicators are used in a new context [32]. Each patient care process will be scored according to the criteria. Scoring will be done by a panel of specialists blinded to the intervention status of the patient. We will calculate the quality score as adherence scores (number of quality criteria met divided by number of applicable criteria expressed as a percentage) as illustrated by Ashton *et al*. [33]. If a criterion is applicable, but no information can be found (applicable, but not answerable), it will be noted ‘not met’ for statistical purposes [34]. Weighting of the criteria based upon clinical importance will not be used, as this adds complexity without adding much to the clinical findings, a finding discussed by Lyons and Payne in 1975 [35]. The scores will be compared between the intervention and control groups.

As secondary outcomes, the quality of the referrals will be evaluated against the developed referral template, to determine if the intervention has led to improved referral quality. It is important to measure referral quality, as the premise in the study is that more information will lead to improved care. In addition, health process outcomes such as waiting time from referral to appointment, number of appointments before a diagnosis is reached, time before treatment is initiated, the application or not of appointment prioritization, and the outcome of any given referral (appointment, return information or referral rejected) will be collected and compared between the groups. Bennett *et al*. used the PPV of a referral as a measure of the appropriateness of the referral [31]. In adapting this concept from glue ear in otolaryngology to a medical department we have defined it as the proportion of the GP referrals that result in a histological diagnosis, diagnostic clarification or change in medical management. We will assess and compare the PPV of referrals in the study groups. Patient experiences will be compared between the intervention and control group. Finally, the possible outcome diagnoses have been grouped according to severity. We will correlate the continuous outcome variable ‘waiting time’ with the grouped severity, to see whether the prioritization in the intervention group is more aligned with disease severity.

### Sample size

Sample size was calculated based upon the primary outcome, and the initial calculation was done without regard for clustering. A change in the quality score of 10% was determined to be clinically interesting. Setting the significance level at 0.05 and using PASS 2008 (NCSS, LLC, Kaysville, UT, USA) for the calculation provides that the study would require 855 patients in the control and 855 in the intervention group, for an 80% power to detect a 10% difference between the groups.

To correct for clustering, the design effect (DE) may be calculate as:

DE = 1 + *ρ*(*m*-1)

where *ρ* denoted the intracluster correlation coefficient (ICC) and *m* is the size of each cluster [36]. No ICC for equivalent designs was identified from literature searches. Reviewing primary care-based interventions from the literature [37-39], an expected ICC ranging from 0.001 to 0.08 does not seem improbable, giving a DE of 1.15 and 12.9, respectively, for a cluster size of 150 patients. Because only 14 GP clinics were available for randomization, further inflation of the number of clusters to achieve higher power was not possible, although this could have been advisable [40].

Based upon a review of patient data at UNN Harstad from 2008, the study is expected to achieve this relatively high inclusion number by recruiting over a two-year period (personal data).

### Blinding

The referring GP cannot be blinded to the trial, as the intervention is actively used by the GP. The patient will not be aware of the intervention, but no active effort has been made to keep the patients blinded. The patients will be mixed with the general caseload to avoid bias in the treatment process at the hospital. For the GPs that use the electronic referral template, this usage will be visible to the hospital doctor in the presentation of the referral letter on the computer, but for the majority of the cases the hospital doctor will be blinded to the intervention status of the patient. The outcome assessors will be blinded.

### Data gathering

Data will be extracted by both automated computer reports (for example, waiting times, number of appointments) and manual chart review (for example, PPV, group of final diagnosis). Data will be collected after the process of care that the referral initiated is completed.

### Statistical methods

We will collect the following baseline characteristics:

• patient age (mean and confidence interval) and sex (number and percentage)

• practice list size (median and interquartile range, or mean and confidence interval if normally distributed)

• referral type - electronic or paper (number and percentage)

• referred by - GP or other doctor (number and percentage).

For the primary outcome (quality score), we will calculate adherence scores as described above and compare between treatment arms. We expect substantial variation in cluster size. Because of the small number of clusters, analysis based upon the cluster level was considered [41]. However, as there is no prior accurate estimate of variation between clusters, weighting for cluster size could not be achieved [41]. To offer increased precision and take into account between-cluster variation, random effects linear regression will be used [42] to generate estimates of intervention effect. It has been suggested that this can be used for studies with as few as 10 clusters [36]. The estimated effect and confidence intervals will be reported. A *P*-value <0.05 will be regarded as statistically significant. Intention to treat analysis will be employed.

The referrals will be scored using a simple scoring system related to the referral templates. Each unit of information specified in the referral template (for example, presence of weight loss specified) will provide one point in the scoring system, with no weighting applied. Scores will be compared between the groups as noted above.

For outcome severity, random effects linear regression will be used, with the severity group score as a categorical variable, and the relationship compared between the intervention and control group.

In the questionnaire, answers noted as ‘not applicable’ or no answer will be counted as missing data. The questionnaire will be scored according to a pre-set scoring system. Scores will be analyzed using the regression technique outlined above. The data will also be analyzed to determine if factors such as self-perceived health, age, gender, and education level have an impact on patient experience.

The trial will be reported according to the CONSORT standards for reporting cluster randomized trials [43].

### Pilot study

No pilot study has been carried out. To ensure acceptability of the intervention, GPs were invited to, and participated in, the development of the referral template. To ensure feasibility, the authors have collected all data specified in the protocol from the 20 patients included first. To ensure an adequate uptake of the intervention, regular reviews of all referrals received at UNN Harstad will be undertaken.

### Ethics

The study will follow the directions in the Helsinki Declaration, and was presented to the Regional Ethical Committee for Medical Research in northern Norway, who determined it not to be within the scope of the Health Research Act (REK NORD 2010/2259). The project has been approved by the Data Protection Official for Research. The study is registered at ClinicalTrials.gov. The trial registration number is NCT01470963. All patients must provide written informed consent.

## Discussion

Transitions of care represent a point of frequent adverse events [44,45]. The referral is the main form of communication in the transition from primary to secondary care [13,46]. Although many referral interventions have been evaluated, there appears to be limited knowledge on how the referral letters affect specialist care. A recent study protocol describes a similar project within mental health care [47], although this study is still ongoing. The primary objective of our study is to assess whether an improved referral letter will lead to a measurable change in the quality of care delivered in a medical department. The aim is to go beyond an assessment of referral quality and waiting times *per se*, and evaluate quality of care and the appropriateness of waiting times and treatment, and, as such, help fill parts of the knowledge gap identified in a Cochrane review on the subject [18].

However, research at the interface between primary and secondary care can be challenging [18,48]. The choice of using an intervention with intuitive content was made to make it acceptable in normal general practice. The assumption underlying this research project is that a referral guideline will increase the amount of information available to the hospital specialist, and that this increase in information will translate into better care.

The cluster design was chosen because randomization with this approach is well suited for interventions implemented at the level of the health care organizational unit [42]. In addition, randomizing at the individual patient level would undermine findings, as the GPs could use the information learned from the referral template in their non-intervention referrals and as such contaminate the data. For similar reasons, the GP clinic, as opposed to the individual GP, was randomized, as contamination between GPs in the same clinic was to be expected.

In choosing a cluster randomized design, we have a design that is less statistically efficient than a standard randomized design. A recent study involving a more complex intervention [49] used randomization at the patient level to avoid this problem. We feel that the dangers of contamination with individual randomization in our design would be so large that results would be difficult to interpret.

In cluster randomized trials, post randomization bias has been identified as a concern [40]. This entails the recruitment of different cohorts in the intervention and control groups, as the patients are recruited after randomization of the clusters. We hope to reduce this by actively recruiting the patients (obtaining signed consent) in conjunction with the hospital appointment, both for the intervention and control groups.

The intervention is intentionally simple to ensure that an effect seen can be attributed to the intervention. However, an intervention at an interface in a complicated health care system can quickly affect the entire process, in ways we have not yet envisaged.

There is also a risk of performance bias as systematic differences in the care may not be due to the intervention, but rather because the doctors will be aware of the study protocol. By ensuring the mixing of cases in normal workloads and, as much as possible, blinding the doctors involved, we hope to minimize this bias. The fact that the care process is being studied may change the behavior of the doctors in general, akin to a Hawthorn effect. This will potentially attenuate the effects of the intervention, as the quality of care may improve for both intervention and control patients.

The authors also recognize that many referrals from primary to secondary care are not made only to identify major pathology. Referrals are also made to reassure the patient, reduce medico-legal risk, obtain a second opinion or for handing over of care [50]. The authors fully appreciate these as valid reasons for referral. We, therefore, do not aim to reduce the number of referrals, but rather assess the effect on hospital care of improved referrals.

This study aims to add to the knowledge regarding the effect of the referral on the patient pathway and quality of care. Simple diagnostic groups have been chosen. If the study can identify benefits from improving referrals in these areas, this may lead the way to further implementations of referral proforma, preferably electronically integrated into the standard software packages. This could improve the overall referral process to enable better care and effective prioritization based upon the need of the individual patients.

## Trial status

The study began including patients in the fall of 2011 and inclusion is planned for approximately two years.

## Abbreviations

GP: general practitioner; PPV: positive predictive value.

## Competing interests

The authors declare that they have no competing interests.

## Authors’ contributions

The idea behind the study was conceived by PCV. All authors participated in the study concept and design. HW is the grant holder. ARB, PCV and HW developed the referral guidelines and outcome measures. All authors reviewed and approved the final version of the manuscript.
